# Global cluster analysis and network visualization in cancer-associated fibroblast: insights from Web of Science database from 1999 to 2021

**DOI:** 10.1186/s40001-023-01527-3

**Published:** 2023-11-29

**Authors:** Dun Yuan, Bo-Wen Zheng, Bo-Yv Zheng, Hua-Qing Niu, Ming-Xiang Zou, Song-Lin Liu, Fu-Sheng Liu

**Affiliations:** 1 Department of Neurosurgery, Xiangya Hospital, Central South University, Changsha, China; 2 National Clinical Research Center for Geriatric Disorders, Xiangya Hospital, Central South University, Changsha, China; 3 Musculoskeletal Tumor Center, Peking University People’s Hospital, Peking University, Beijing, 100044 China; 4 Department of Orthopedics Surgery, General Hospital of the Central Theater Command, Wuhan, 430061 China; 5 Department of Ophthalmology, The Second Affiliated Hospital of Zhengzhou University, Zhengzhou, 450014 Henan China; 6 Department of Spine Surgery, The First Affiliated Hospital, University of South China, Hengyang, 421001 China; 7 Department of Spine Surgery, The Second Xiangya Hospital, Central South University, Changsha, 410011 China

**Keywords:** Global research trend, Bibliometric analysis, Cancer-associated fibroblasts, Tumor-associated fibroblasts, VOSviewer, Visualized analysis

## Abstract

**Background:**

A scientific and comprehensive analysis of the current status and trends in the field of cancer-associated fibroblast (CAF) research is worth investigating. This study aims to investigate and visualize the development, research frontiers, and future trends in CAFs both quantitatively and qualitatively based on a bibliometric approach.

**Methods:**

A total of 5518 publications were downloaded from the Science Citation Index Expanded of Web of Science Core Collection from 1999 to 2021 and identified for bibliometric analysis. Visualized approaches, OriginPro (version 9.8.0.200) and R (version 4.2.0) software tools were used to perform bibliometric and knowledge-map analysis.

**Results:**

The number of publications on CAFs increased each year, and the same tendency was observed in the RRI. Apart from China, the countries with the largest number of publications and the most cited frequency were mainly Western developed countries, especially the USA. *Cancers* was the journal with the largest number of articles published in CAFs, and Oncology was the most popular research orientation. The most productive author was Lisanti MP, and the University of Texas System was ranked first in the institutions. In addition, the topics of CAFs could be divided into five categories, including tumor classification, prognostic study, oncologic therapies, tumor metabolism and tumor microenvironment.

**Conclusions:**

This is the first thoroughly scientific bibliometric analysis and visualized study of the global research field on CAFs over the past 20 years. The study may provide benefits for researchers to master CAFs' dynamic evolution and research trends.

**Supplementary Information:**

The online version contains supplementary material available at 10.1186/s40001-023-01527-3.

## Introduction

The biological characteristics of the tumor, such as occurrence, development, invasion and metastasis, are not only closely related to tumor cells themselves, but also strongly associated with the environment in which tumor cells are located, namely tumor microenvironment (TME) [1]. TME plays an important role in tumor growth, drug resistance and metastasis by providing corresponding support for tumor cells as “soil” [2]. TME includes various cells, such as tumor cells, immune cells, endothelial cells, and cancer-associated fibroblasts (CAFs), as well as various matrix components surrounding the extracellular surface [3–5]. Among these cells, CAFs, as the most abundant stromal cells in the tumor microenvironment, play an important role in the growth, invasion and metastasis of tumor cells [6]. Previous study had shown that the volume of CAFs in solid tumors account for up to 80% [7].

Studies have shown that CAFs are complex heterogeneous tumor cell populations, which may be related to their various cell sources [8]. According to histological types, CAFs mainly come from six kinds of cells: fibroblasts in normal tissues; bone marrow-derived fibroblasts; mesenchymal stem cells; epithelial–mesenchymal transition; endothelial–mesenchymal transition; pericytes, smooth muscle cells and adipocytes [8–11]. CAFs run through the whole process of tumorigenesis and play an important role in tumor evolution and derivation [12]. They can benefit tumor cells from the following aspects: promote the growth and proliferation of tumor cells [13, 14]; assist tumor-related angiogenesis [15, 16]; accelerate invasion and metastasis of tumor cells [17]; elicit resistance to certain anti-tumor drugs [18, 19]. CAFs promote tumor progression by secreting a large number of cytokines and remodeling extracellular matrix, and play a key role in building a microenvironment conducive to tumor occurrence, angiogenesis, diffusion and metastasis [20]. Therefore, the in-depth study of CAFs will be of great significance to the treatment, prognosis and drug development of tumors.

With the deepening of research, it is found that the role of CAFs is more and more extensive. It is of great significance to analyze the previous literature to understand the progress of CAFs research and predict the research trend. Bibliometric analysis, which analysis the qualitative and quantitative information of a given topic from the published literature using mathematical and statistical methods, can elucidate the research focus and emerging trends [21, 22]. Compared to other retrospective studies, bibliometric research has potential advantages such as more objective data collection, larger sample size, lower cost-effectiveness, and the ability to analyze the relationship between researchers. The global development trends of CAFs research, however, have not been fully analyzed. To make up for this deficiency, this study would use the method of bibliometrics to analyze and visualize of the relevant information in previous literature, such as countries, year of publications, institutions, journals, funs, authors and keywords, and summarized the hotspots and development trends of current research, which provides researchers with a more intuitive understanding and has certain guiding and reference significance for future research.

## Materials and methods

### Data source and search strategy

Electronic searches of the Web of Science (WoS) database, which is a large, comprehensive, multidisciplinary and authoritative database internationally recognized and suitable for bibliometric analysis, were performed from inception to August 15, 2022, to obtain the literature of interest. The following terms used for searching were “cancer-associated fibroblast*” OR “tumor-associated fibroblast*” OR “cancer-related fibroblast*” OR “tumor-related fibroblast*”. The database was restricted to the Web of Science Core Collection (WoSCC), and the language was limited to English, and the type of articles was limited to research articles and reviews. A total of 5518 records were marked and selected as “full record with cited references” exported in the “plain text file” format and renamed following the convention “download ∗ .txt” to ensure proper compatibility with the VOSviewer software. We accessed WoS database via the electronic library of Central South University, with access rights provided by the university. Two authors (FSL and BWZ) independently conducted the literature search and review. Any dispute would be resolved by consensus.

### Eligibility criteria

**Fig. 1:**
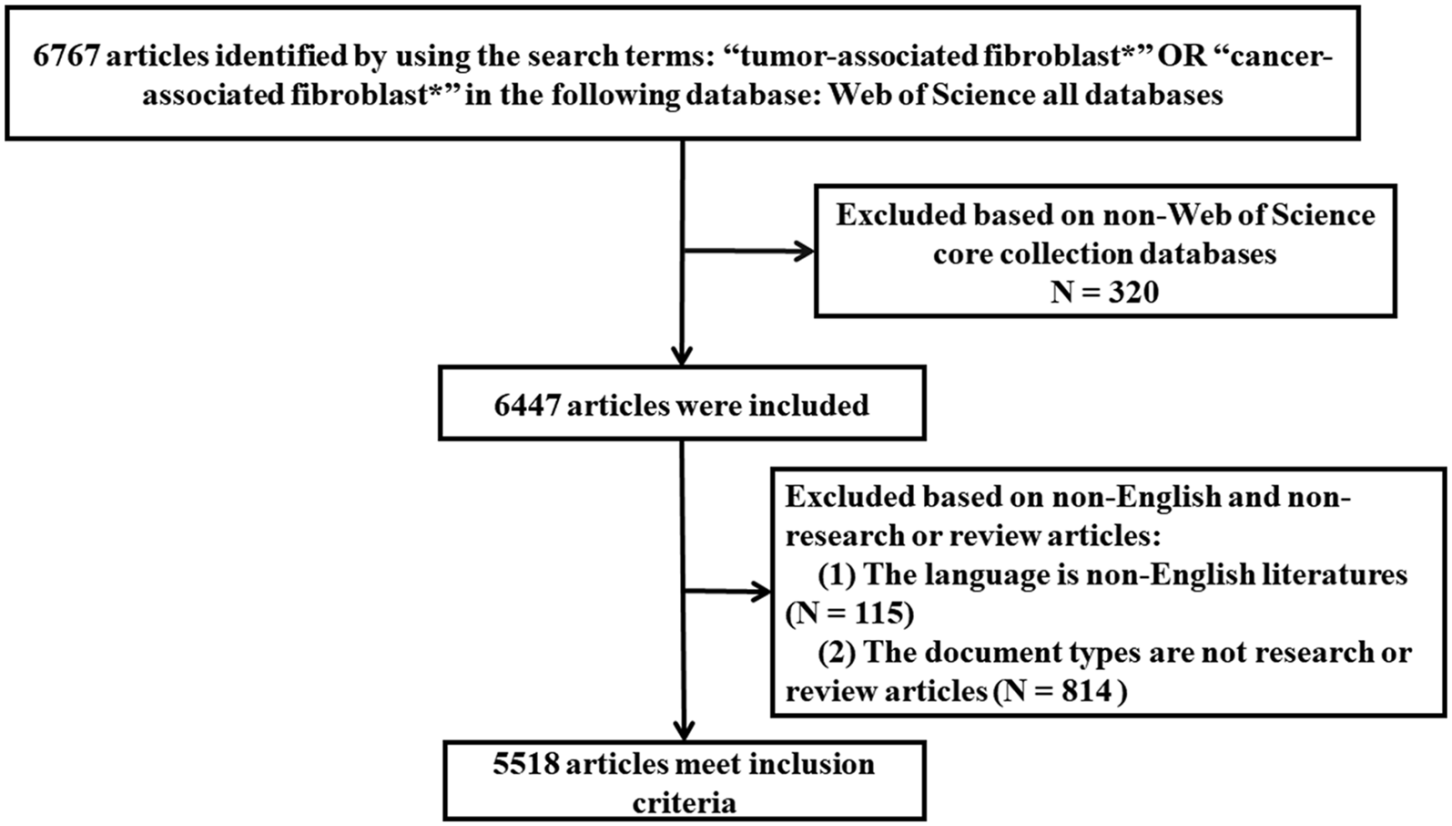
Flowchart of the screening process

As shown in Fig. 1, the inclusion criteria were as follows: (1) a clear correlation with CAFs; (2) research or review articles with CAFs; (3) theme focus on CAFs. The exclusion criteria were as follows: (1) content not related to CAFs; (2) the articles were not written in English; (3) the type of articles was case report, meeting, letter, comment and abstract, etc.

### Data extraction

From the WoSCC database, we collected and downloaded the data and saved them as.txt files, including authors’ names, article titles, abstracts, keywords, year of publications, the number of citations, journal titles, institutions, funds, references, countries and languages, etc. The “analyze results” and “citation report” functions in the WoS database's online analysis feature were utilized to conduct a comprehensive analysis of the aforementioned content. Then, the data were processed independently by two authors according to the inclusion and exclusion criteria. Finally, the processed data were imported into the corresponding software (VOSviewer, Origin and R Language) for further analysis.

### Bibliometric and visualized analysis

Based on the information provided by the WoS database, which could provide detailed information and was the preferred database for bibliometrics study, we analyzed the annual publication volume and relative research interest (RRI) of CAFs, and predicted the number of publications in the next years. RRI means that the number of papers published in a one field accounts for the number of papers published in the all fields per years, which was a good indicator of the research interest in a particular field. Then, the number of publications in different countries/regions was visually displayed in the form of a world map. In addition, we specifically analyzed the top 10 countries/regions of the total publications. Total citations, average citations and H-index were used to analyze the quality of paper publications. Finally, the top 25 countries/regions, journals, research orientations, authors, institutions and funds were analyzed. The above analyses were performed using OriginPro (version 9.8.0.200) and R (version 4.2.0) software.

Visualized analysis was performed using VOSviewer (version 1.6.16) and Pajek (version 5.16) software. We constructed visualization maps to obtain the relationships between research components using different analytical features of the VOSviewer software. These results are presented in network diagram, which is composed of clusters in different colors with nodes and link-lines. The size of node often indicates importance or influence or the frequency of the co-occurrence or co-citation of the studied objects. The thickness of the link-lines usually reflects the strength of the association or similarity between nodes. The colors of nodes and link-lines often represent different attributes or categories. To comprehensively understand the relationship between the literature, we performed co-citation analysis, co-authorship analysis and co-occurrence analysis. The research hotspots and trends were summarized through the keywords co-occurrence analysis.

## Results

### Global contribution to the research of cancer-associated fibroblasts

**Fig. 2:**
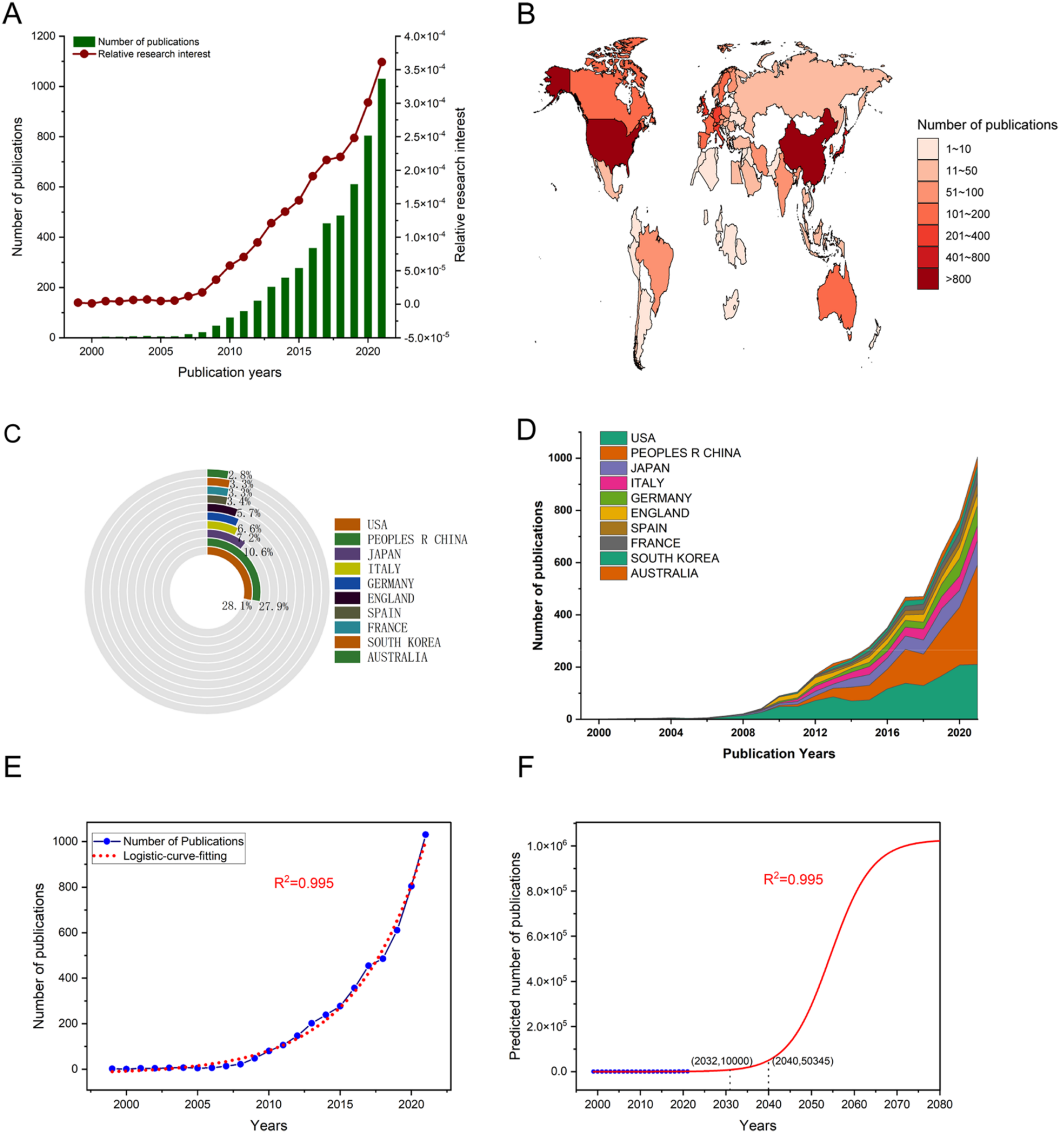
**A** The global number (blue bars) and relative research interests (red curve) of publications related to cancer-associated fibroblasts (CAFs) research from 1999 to 2021. **B** Distribution of CAFs research from 1999 to 2021 in world map. **C** The sum of publications CAFs research from the top 10 countries and regions. **D** The annual number of publications in the top 10 most productive countries from 1999 to 2021. **E** and **F** Model logistic fitting curve of global trends in publications related to CAFs research per year (*R*^2^ = 0.995, (2032, 10,000) indicates that the total publications will up to 10,000 in year of 2032), (2040, 50,345) indicates that the total publications will up to 50,345 in year of 2040)

A total of 6767 papers matched our initial search strategy from the WoS database. After screening, there were 5518 pieces of literature, which were published between 1999 and 2022, finally included in our study for further analysis (Fig. 1). As shown in Fig. 2A, the number of publications showed an increasing trend year by year, from 2 in 1999 to 1031 in 2021, and the same trend was observed in the RRI. These results indicated that the academic community was paying more and more attention to CAFs. Subsequently, we performed curve fitting based on the present published CAFs articles according to the previous article [23] and predicted the publications in the future. A good fitting curve (logistic regression model) was obtained as shown by the red dotted line in Fig. 2E (*R*^2^ = 0.995). In the predictive model, it can be seen that the number of publications with CAFs will reach 10,000 in 2032, and in 2040, the number of articles will reach a staggering 50,345 (Fig. 2F). Moreover, we constructed separate logistic regression models for publications in developed and developing countries, and obtained some noteworthy results (Additional file 1: Fig. S1). Specifically, our findings indicate that at present, developed countries (Additional file 1: Fig. S1, B) have a greater publication volume and growth rate than developing countries (Additional file 1: Fig. S1C, D), but in the future, developing countries are expected to surpass developed countries in terms of publications. This observation may suggest that developing countries are allocating more resources to the medical field as their economies advance. A total of 77 different counties/regions contributed to the 5518 articles of CAFs. From the heat map of the world map (Fig. 2B), we could see that the USA and China were in the first tier with the highest number of published articles, 1549 and 1541, respectively. Japan and Italy were close behind in the second tier, with 586 and 400 articles published, respectively. In addition, we analyzed the top 10 countries with the most published articles, as well as the details of their publications in each year. As shown in Fig. 2C, the number of papers published on CAFs in the USA accounts for 28.1%, ranking first. Followed by China, accounting for 27.9%. Compared with the former two countries, decreased significantly in Japan, Italy and Germany, which were 10.6%, 7.2% and 6.6%, respectively. Before 2008, the top 10 most productive countries published a few articles every year, and then gradually increased. The USA had always maintained the leading position in the annual number of publications, until after 2019, when it was overtaken by China (Fig. 2D). These data indicated that China had funded and produced more and more in CAFs research in recent years.

**Fig. 3:**
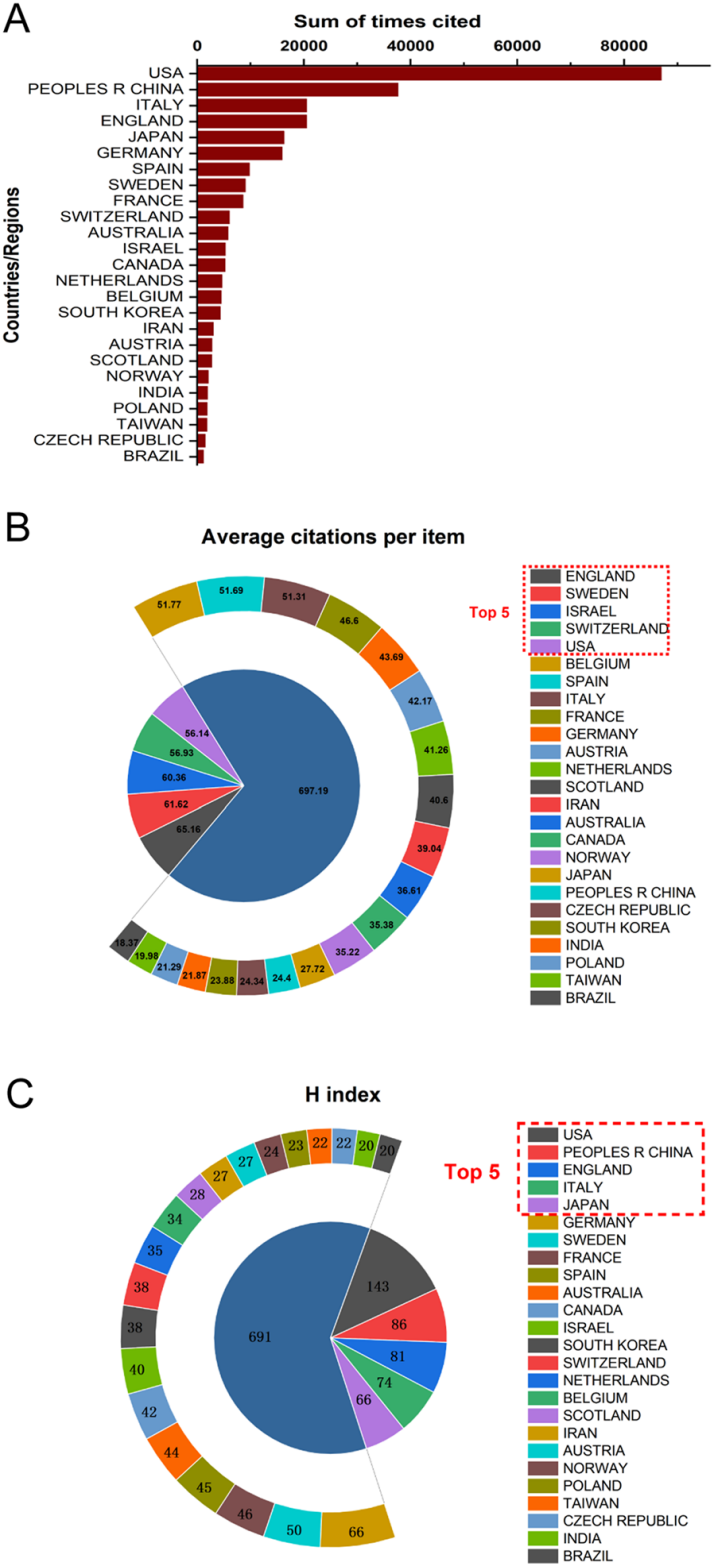
**A** The top 25 countries/regions of total citations related to CAFs research from 1999 to 2021. **B** The top 25 countries/regions of the average citations per publication related to CAFs research from 1999 to 2021. **C** The top 25 countries/regions of the publication H-index related to CAFs research from 1999 to 2021

Regarding total citation times, Fig. 3A shows the total citation frequency of top 25 countries, with the USA (86,967), China (37,608), Italy (20,525), England (20,524) and Japan (16,242) ranked in the top five. In terms of the average citation frequencies, the times of England ranked first with 65.16, followed by Sweden (61.62), Israel (60.36), Switzerland (56.93) and USA (56.14) (Fig. 3B). As shown in Fig. 3C, the USA (143), China (86), England (81), Italy (74) and Japan (66) ranked in the top five of the H-index, which indicated that these countries were highly influential and productive on CAFs research.

### Analysis of global publications

**Fig. 4:**
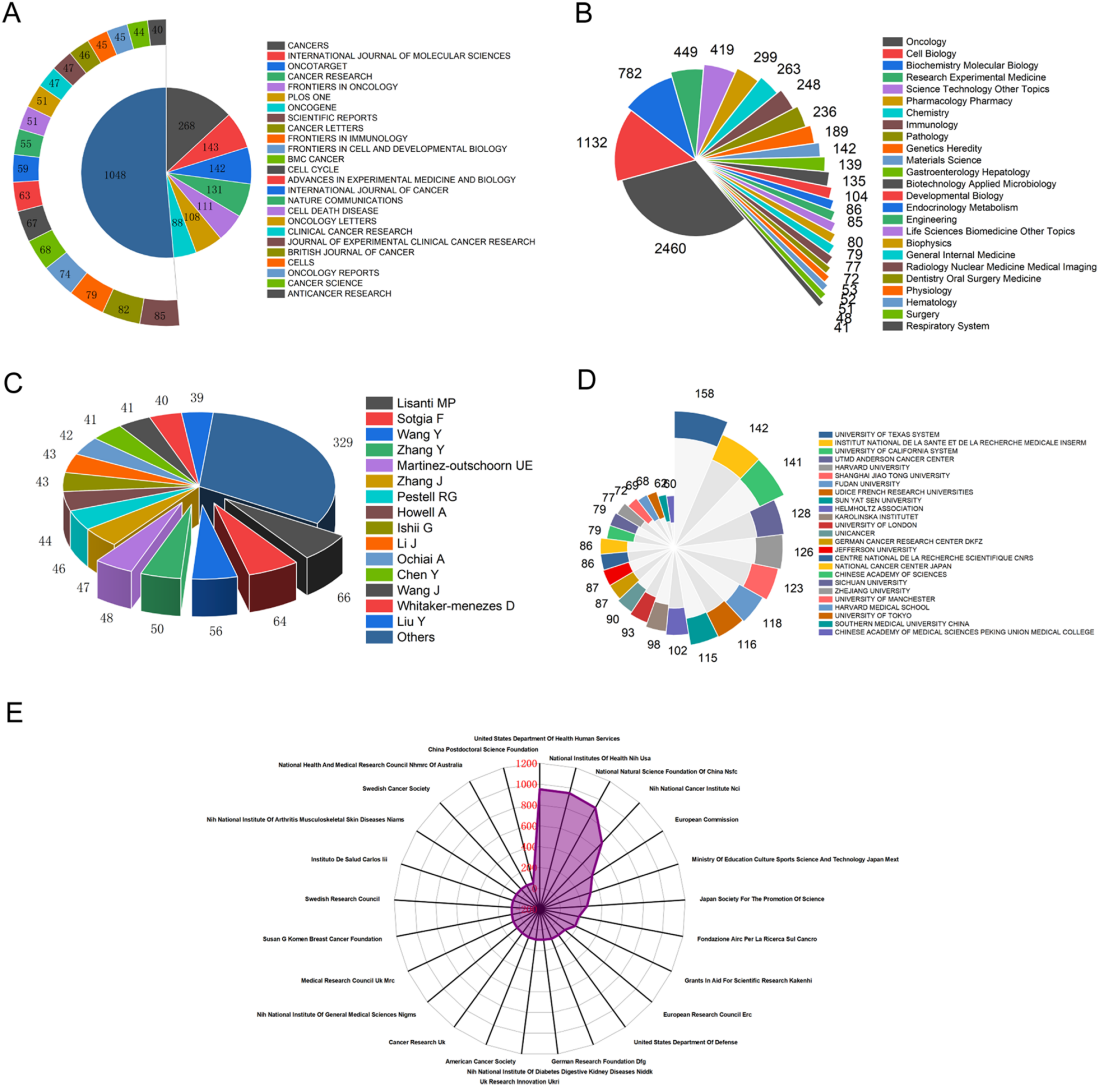
**A** The top 25 journals with most publications related to CAFs research from 1999 to 2021. **B** The top 25 research orientations with most publications from 1999 to 2021. **C** The top 25 authors related to CAFs research from 1999 to 2021. **D** The top 25 institutions with most publications related to CAFs research from 1999 to 2021. **E** The top 25 funds with most publications related to CAFs research from 1999 to 2021

To investigate the active leading journals in CAFs, we executed a journal distribution analysis of publications. The top 25 journals with the most publications are shown in Fig. 4A, with published 2039 articles, accounting for 36.95% of the total publications on CAFs. At the top of the list was the journal of *Cancers* (IF = 6.575, 2021), with 268 papers; followed by *International Journal of Molecular Sciences* (IF = 6.628, 2021), with 143 papers. There are 142 articles in *Oncotarget* (IF = 5.168, 2016), 131 in *Cancer Research* (IF = 13.312, 2021) and 111 in *Frontiers in Oncology* (IF = 5.738, 2021). In terms of research orientations, Fig. 4B lists the top 25 research directions with the most publications. The top 5 popular research areas in the first tier were oncology (2460 articles), cell biology (1132 articles), biochemistry molecular biology (782 articles), research experimental medicine (449 articles) and science technology other topics (419 articles).

For prolific authors analysis, the top 25 authors are listed in Fig. 4C. They contributed 1039 publications, accounting for 18.83% of the total publications on CAFs. As shown in Fig. 4C, Lisanti MP was the most productive author with 66 articles. Followed by Sotgia F with 64 articles, Wang Y with 56 articles, Zhang Y with 50 articles and Martinez-outschoorn UE with 48 articles. Regarding the institutions analysis, the top 25 fruitful institutions are listed in Fig. 4D. Top of list was University of Texas System with 158 publications, then was Institut National de la Sante et de la Recherche Medicale with 142 publications and University of California System with 141 publications.

The radar chart showed the top 25 funding institutions of CAFs-related articles. As shown in Fig. 4E, there were 951 studies funded by United States Department of Health Human Services ranked first. Followed by National Institutes of Health (NIH, USA), which funded 949 publications. While the National Natural Science Foundation of China (NSFC, China) funded 909 studies ranked third. In addition, the National Cancer Institute (NCI, USA, 673 publications) and European Commission (396 publications) had also funded many research projects.

### Bibliographic coupling analysis

**Fig. 5:**
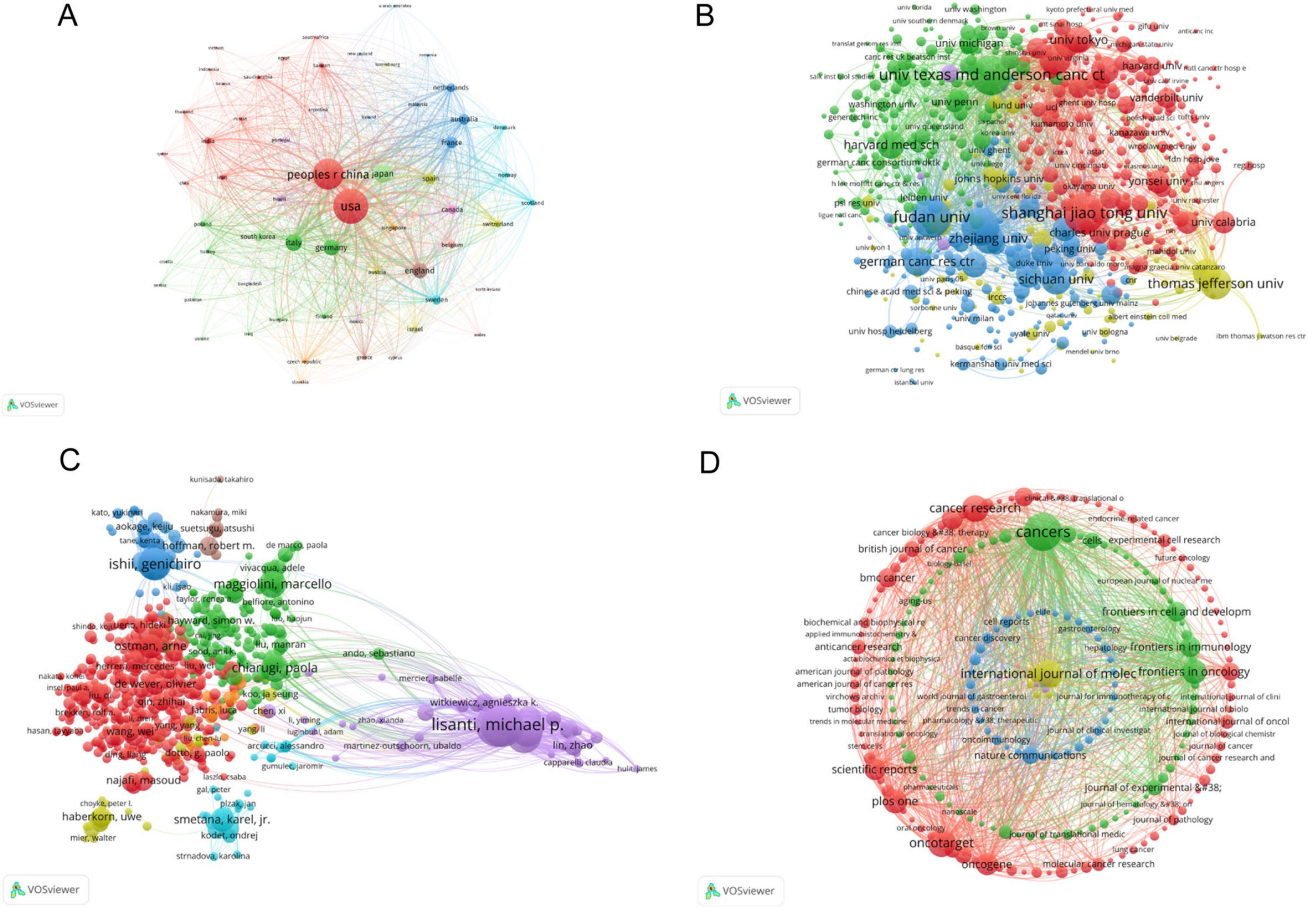
**A** Mapping of the 60 countries related to CAFs research from 1999 to 2021. **B** Mapping of the 640 institutions related to CAFs research from 1999 to 2021. **C** Density visualization of the 765 authors related to CAFs research from 1999 to 2021. **D** Mapping of the 216 identified journals related to CAFs research from 1999 to 2021

Bibliographic coupling analysis was used to explore the commonality and correlation between documents. In the analysis of counties, the publications were selected with the maximum number of countries no more than 25 per documents and the minimum number of documents per countries at least 5. In this visual analysis (VOSviewer), a total of 80 countries were included, of which 60 countries met the above threshold. As shown in Fig. 5A, the top 5 countries that showed the largest total link strength on CAFs were USA (total link strength = 4,130,915 times), China (total link strength = 3,345,710 times), Japan (total link strength = 1,512,480 times), Italy (total link strength = 1,246,659 times) and Germany (total link strength = 1,170,891 times).

In terms of organizations analysis, the data processing principle was the same as above. The total of 4565 organizations were selected, of which 640 organizations met the thresholds. Among the 640 institutions, Univ Texas MD Anderson Canc Ctr with the total link strength 589,123 times ranked first, followed by Fudan Univ with the total link strength 505,090 times and Sun Yat Sen Univ ranked third with the total link strength 460,333 times (Fig. 5B).

Regarding the authors bibliographic coupling analysis, there were 29,274 authors contributing the publications, of which 765 met the threshold. As shown in Fig. 5C, the top 5 prolific authors with the largest total link strength were listed below: Lisanti, Michael P (total link strength = 374,719 times), Sotgia, Federica (total link strength = 369,005 times), Martinez-outschoorn, Ubaldo E (total link strength = 304,269 times), Pestell, Richard G (total link strength = 291,015 times) and Howell, Anthony (total link strength = 271,710 times).

Bibliographic coupling analysis on the names of journals is shown in Fig. 5D. Overall, 896 different journals published the 5518 articles and 216 journals met the thresholds (the minimum number of documents per journal at least 5). The top 5 prevalent journals that showed the most considerable total link strength were listed as follows: *Cancers*, *International Journal of Molecular Sciences*, *Frontiers in Oncology*, *Cancer Research* and *Oncotarget*, with the total link strength 640,457 times, 293,342 times, 229,081 times, 199,249 times and 196,694, respectively.

Furthermore, we also conducted keyword co-occurrence analyses on the top three countries (USA, China, and Japan) and institutions (University of Texas System, Institut National de la Sante et de la Recherche Medicale INSERM, and University of California System) with the highest publication volume. The visualized results (Additional file 2: Fig. S2, Additional file 3: Fig. S3) indicate that the USA has conducted more research on pancreatic cancer, prostate cancer, breast cancer, and related disease mechanisms (such as autophagy, oxidative stress) and has shown a trend toward specific therapies, such as immunotherapy (Additional file 2: Fig. S2A, 2B). China has conducted more research on lung cancer, gastric cancer, colorectal cancer, and breast cancer, and is also optimistic about the prospects of immunotherapy (Additional file 2: Fig. S2C, D). Japan has focused more on gastric cancer, colorectal cancer, and pancreatic cancer, and has also shown an increased interest in immunotherapy in recent years, paralleling the above two countries (Additional file 2: Fig. S2E, F). Among the top three research institutions, two are from the USA, the University of Texas System, which has conducted more research on pancreatic cancer and ovarian cancer (Additional file 2: Fig. S3A, B), and the University of California System, which has focused more on pancreatic cancer and breast cancer (Additional file 3: Fig. S3E, F). The second-ranked Institut National de la Sante et de la Recherche Medicale INSERM from France has conducted more research on pancreatic cancer (Additional file 3: Fig. S3C, D). These three institutions have shown a significant interest in immunotherapy in recent years.

### Co-citation analysis of authors, journals, and references

**Fig. 6:**
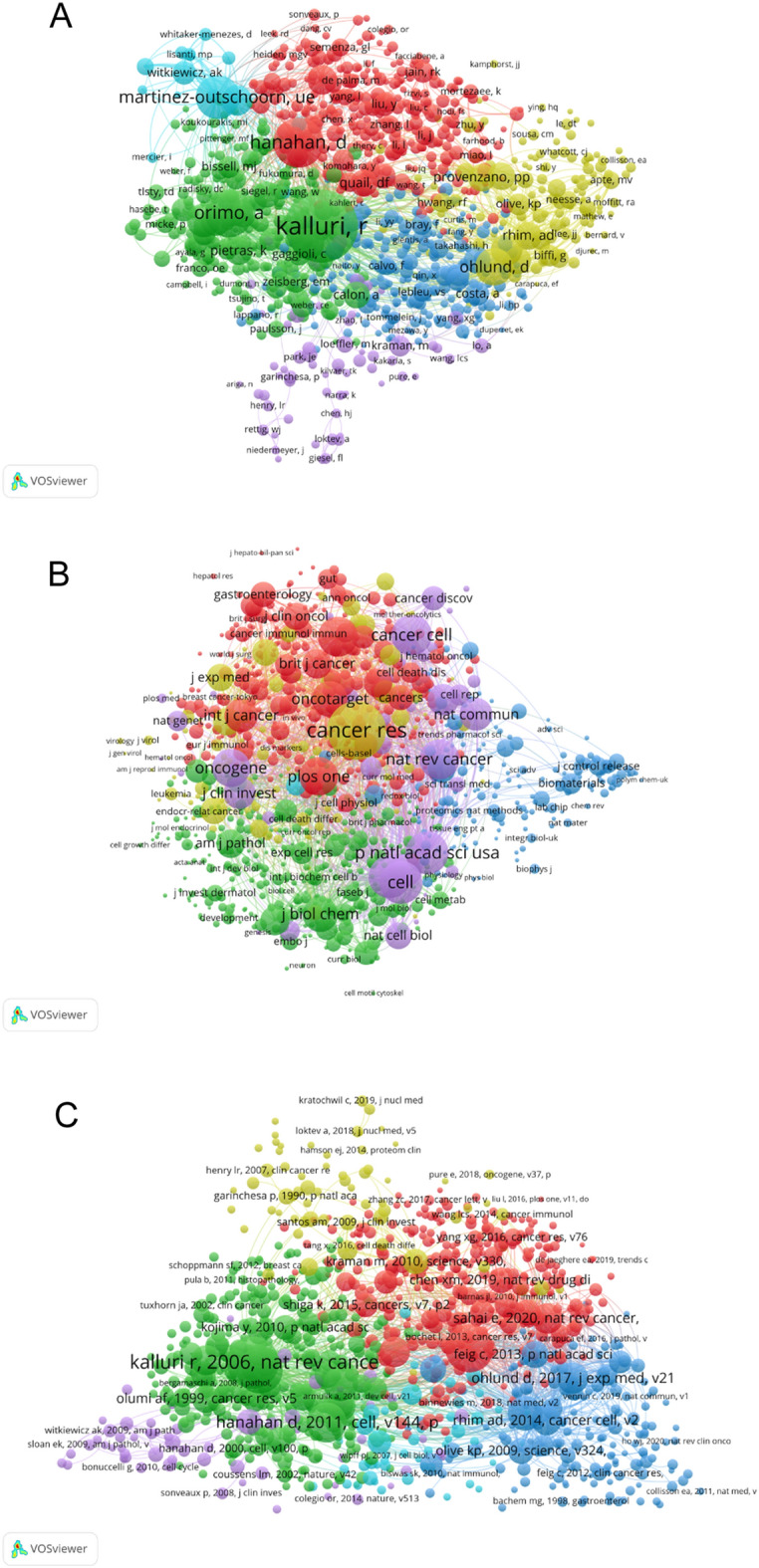
**A** Mapping of the co-cited authors. (The 3029 points with different colors represent the 3029 cited references.) **B** Mapping of the co-cited journals. (The 1000 points with different colors represent the 1000 identified journals.) **C** Mapping of the co-cited references. The point sizes represent the citation frequency. The line between different points indicates that they were cited in one paper. The shorter the line, the closer the link between two papers. The same color of the points represents the same research area they belong to

To explore the academic relationships of authors, journals and references, the co-citation analysis was performed by VOSviewer software. In terms of conditions, the publications were selected with the minimum number of citations at least 20 each author. A total of 109,478 authors were screened, of which 3029 authors met the threshold. As shown in Fig. 6A, the top 5 leading authors with the largest total link strength were Kalluri, R (total link strength = 65,457 times), Hanahan, D (total link strength = 37,407 times), Orimo, A (total link strength = 35,704 times), Martinez-Outschoorn, UE (total link strength = 31,167 times) and Ohlund, D (total link strength = 30,908 times).

Regarding the co-citation analysis of journals, Fig. 6B lists 1000 journals that met the minimum number of 20 citations per journal. *Cancer Research* (total link strength = 2,198,651 times), *Proceedings of the National Academy of Sciences of The United States of America* (total link strength = 1,122,696 times), *Cell* (total link strength = 1,084,173 times), *Nature* (total link strength = 1,080,109 times) and *Cancer cell* (total link strength = 946,971 times) were the top 5 journals with the largest total link strength.

In addition, Fig. 6C clearly shows the co-citation analysis of the references and the data processing principle was the same as above. The top 5 references with the largest total link strength were listed as follows: kalluri r, 2016, nat rev cancer, v16, p582 (total link strength = 21,009 times), orimo a, 2005, cell, v121, p335 (total link strength = 19,719 times), kalluri r, 2006, nat rev cancer, v6, p392 (total link strength = 19,591 times), ozdemir bc, 2014, cancer cell, v25, p719 (total link strength = 16,769 times) and erez n, 2010, cancer cell, v17, p135 (total link strength = 14,677 times).

### Co-authorship analysis of author, country, and institutions

**Fig. 7:**
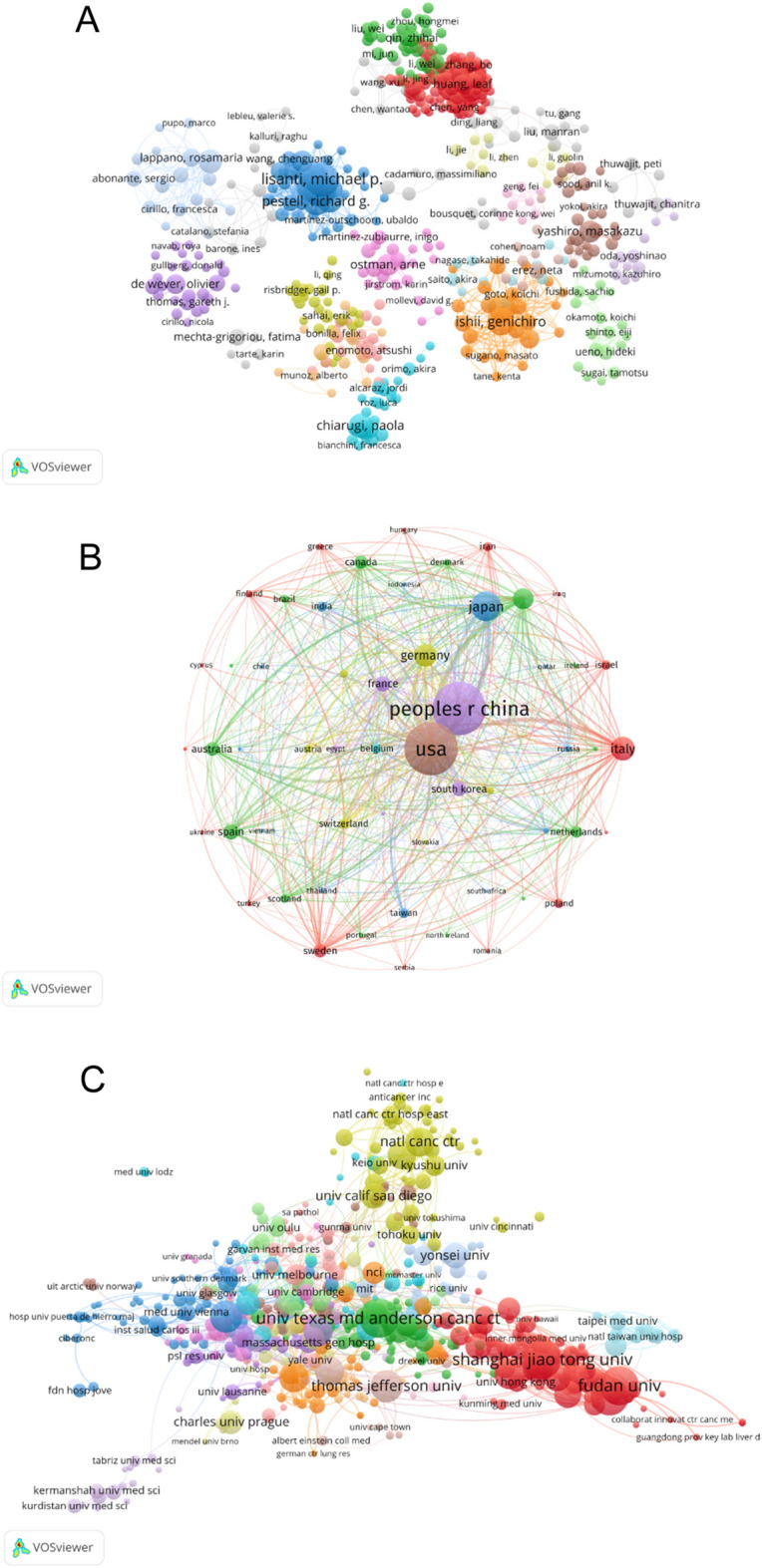
**A** Mapping of the 765 authors co-authorship analysis related to CAFs research from 1999 to 2021. **B** Mapping of the 60 countries co-authorship analysis related to CAFs research from 1999 to 2021. **C** Mapping of the 640-organizations co-authorship analysis related to CAFs research from 1999 to 2021

To explore the relationships of authors, countries and institutions based on the total number of coauthored publications, the co-authorship analysis was performed by VOSviewer with the minimum number of 5 documents of per author. As shown in Fig. 7A, 765 authors met the thresholds. The top 5 authors with the largest total link strength were shown below: Lisanti, Michael P (total link strength = 462 times), Sotgia, Federica (total link strength = 456 times), Pestell, Richard G (total link strength = 377 times), Martinez-Outschoorn, Ubaldo E (total link strength = 373 times) and Howell, Anthony (total link strength = 358 times).

In terms of co-authorship analysis of countries, a total of 80 countries were screened and 60 countries met the thresholds with the minimum number of 5 documents of per country. The top 5 productive countries with the most considerable total link strength are listed in Fig. 7B. USA ranked first (total link strength = 1062 times), England ranked second (total link strength = 401 times), China ranked third (total link strength = 392 times), Germany ranked fourth (total link strength = 372 times) and Italy (total link strength = 339 times).

A total of 4565 organizations were screened in the co-authorship analysis of institution, of which 640 organizations met the thresholds with the minimum number of 5 documents of per organization. The top 5 institutions with the largest total link strength were listed as follows: Univ Texas MD Anderson Canc Ctr (total link strength = 271 times), Harvard Med Sch (total link strength = 210 times), German Canc Res Ctr (total link strength = 208 times), Karolinska Inst (total link strength = 184 times) and Univ Tokyo (total link strength = 151 times) (Fig. 7C).

### Co-occurrence analysis of author keywords

**Fig. 8:**
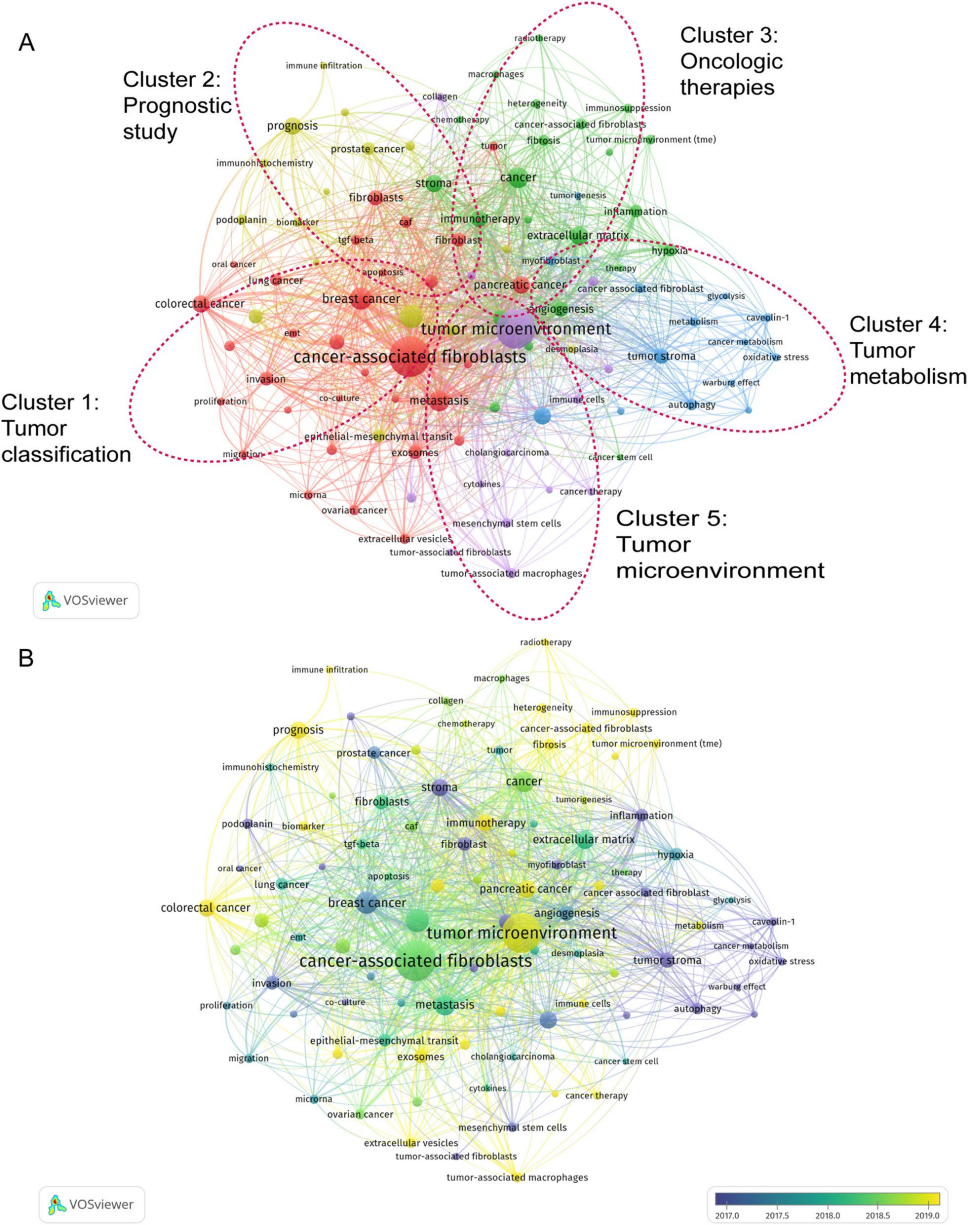
**A** Mapping of keywords in the research related to CAFs research from 1999 to 2021; the frequency is represented by point size and the keywords of research fields are divided into five clusters: tumor classification (red), prognostic study (yellow), oncologic therapies (green), tumor metabolism (blue), and tumor microenvironment (purple). **B** Distribution of keywords according to the mean frequency of appearance; keywords in yellow appeared later than those in blue

Towards better understanding of current research state and hotspots, we performed co-occurrence analysis of author keywords based on VOSviewer software. There were 7552 author keywords extracted from the publications and 97 keywords repeated occurrence at least 30 times were selected and analyzed. As shown in Fig. 8A (network visualization), the 97 identified author keywords were roughly divided into 5 clusters, all of which were shown in different colors. Cluster 1 with red color focused on tumor classification, of which “breast cancer” occurrences 299 times, “pancreatic cancer” occurrences 178 times, “colorectal cancer” occurrences 176 times, “lung cancer” occurrences 90 times and “ovarian cancer” occurrences 65 times; Cluster 2 (yellow) focused on prognostic study with “prognosis” occurrences 174 times; Cluster 3 (green) focused on oncologic therapies, of which “immunotherapy” occurrences 166 times, “chemotherapy” occurrences 35 times, “radiotherapy” occurrences 33 times and “chemoresistance” occurrences 68 times; Cluster 4 (blue) focused on tumor metabolism with “metabolism” occurrences 50 times; Cluster 5 (purple) focused on tumor microenvironment, of which “tumor microenvironment” occurrences 921 times, “cancer stem cells” occurrences 60 times, “immune cells” occurrences 56 times, “cytokines” occurrences 43 times and “mesenchymal stem cells” occurrences 61 times.

The author keywords were given different color according to the time of appearance, which could visually show the hotspots of recent research. As shown in Fig. 8B (overlay visualization), the more yellow the color in the figure, the more recent the keywords occurrence. Figure 8B indicates that the following keywords have recently been prominent in the literature: exosomes, extracellular vesicles, tumor-associated macrophages, immunosuppression, immunotherapy, metabolism, and tumor microenvironment. These results demonstrate current research directions and hot topics that may continue to be prominent in the coming years.

## Discussion

In this study, we performed visualized analyze of the publications of CAFs from 1999 to 2021 using bibliometric methods. We found that the publications on CAFs were increasing year by year, and the same tendency was observed in the RRI. Apart from China, the countries with the largest number of publications and the most cited frequency were mainly western developed countries, especially the USA. *Cancers* was the journal with the largest number of articles published in CAFs, and Oncology was the most popular research orientations on it. The most productive author was Lisanti MP and the University of Texas System was ranked first in the institutions. In addition, the topics of CAFs could be divided into five categories, included tumor classification, prognostic study, oncologic therapies, tumor metabolism and tumor microenvironment. Recent studies had mainly focused on exosomes, tumor-associated macrophages, immunotherapy, metabolism and tumor microenvironment. In a word, this present study introduced in detail the characteristics, distribution, authors network relationship, research direction and hotspots of CAFs, which would be conductive to a comprehensive understanding of this field.

Research on CAFs is experiencing a rapid surge and is gaining increasing attention from the academic community. As illustrated in Fig. 2A, the number of publications related to CAFs has increased almost linearly since 2009, indicating that this field will remain a research hotspot in the near future, providing reliable support for scholars who wish to invest in this area. The USA leads in CAFs research with the highest number of publications (Fig. 2B, C), and the most total citations (Fig. 3A). The number of publications per year has been gradually increasing (Fig. 2D), and the USA ranks first in H-index and fifth in average citation, indicating that the country has made a significant contribution to CAFs research with numerous high-quality papers. This is inseparable from the investment in this field made by the USA, as evidenced by the funding support in Fig. 4E. China is the second-largest country in terms of publications, total citations, and H-index related to CAFs. The number of publications per year has increased sharply after 2017, exhibiting a trend that surpasses that of the USA, indicating that China has also made outstanding contributions to this area. The H-index of CAFs-related papers published in China ranks high, while the average citation ranks low, indicating that the quality of the papers varies significantly. Notably, the England ranks first in average citation and third in H-index, indicating that the published papers have high quality and good research value. Overall, these findings suggest that CAFs research is a global effort, with different countries making significant contributions to this field.

The past decade has seen the unprecedented growth of CAFs research in many fields, as shown in Fig. 8B, especially in breast cancer, pancreatic cancer, colorectal cancer, gastric cancer, lung cancer and ovarian cancer, etc. The reason may be that CAFs was closely related to the occurrence, development, metastasis and drug resistance of tumors, and the related research of CAFs was getting deeper and more mature. In addition, the growth of scientific literature usually has certain rules, the common one is the S-shaped logical curve growth pattern, which is shown as slow growth at first, then rapid growth in exponential form, and finally slow increase until it tends to be stable. In terms of the current trend in the number of articles published, CAFs research was still in a slow growth phase. As shown in Fig. 2F, the logistic curve fitted the annual publications of articles very well, and predicted the publications in the future. In the recent predicted results, the number of publications may be relatively accurate, but for the long-term results, more data need to be provided to adjust the predicted model. After all, nearly 800,000 articles of CAFs were published in 2060, which was an exaggeration. The translation of the text into English is: the deviation of this prediction result may be related to the fact that the number of publications in different countries belongs to different prediction models. When modeling the number of publications of developed countries and developing countries separately, it is found that the prediction model of developed countries is more in line with the actual situation compared with developing countries. This may be due to the extreme imbalance between publications in China and those in other developing countries. Which also indirectly indicates that there is bias in data analysis and further stratification is needed to continuously improve the prediction model.

As an important component of tumor microenvironment, CAFs may be involved in various stages of tumor pathogenesis and evolution. Firstly, CAFs could promote the growth and proliferation of tumor cells [24–27]. Wen et al. [24] studies had shown that TGF-β1 could promote PGP9.5 expression in CAFs to enhance colorectal cancer cell proliferation via the ERK1/2 and PI3K signaling pathways. Similar results have been found in different tumors, as described by Xie et al. [27], they claimed that CAFs secrete hypoxia-induced serglycin to facilitate head and neck squamous cell carcinoma tumor cells growth through the Wnt/β–catenin pathway activated. In addition, Zheng et al. [13] showed that miR-224-5p in CAFs-derived extracellular vesicles promoted the proliferation of colorectal cancer in vitro and in vivo studies. These results indicated that CAFs was closely related to tumor growth. Secondly, CAFs promoted tumor angiogenesis [15, 28–31]. Unterleuthner et al. [15] showed that CAFs-derived WNT2 increased the angiogenesis in colorectal cancer via secreting proteins associated with angiogenesis function, such as IL-6, G-CSF, and PGF. Wan et al. [29] studies revealed that FOSL2 facilitated VEGF-independent angiogenesis via activating Wnt5a in breast CAFs in vivo study. In addition, after co-culturing metastatic pancreatic cancer cells with CAFs, Pausch et al. [32] found that CAFs were angiogenesis increased in the co-culture medium. They also found that angiogenic factors were over-expressed and their expression only increased in CAFs. These evidences are sufficient to prove the important role of CAFs in promoting tumor angiogenesis. Thirdly, CAFs accelerated the invasion and metastasis of tumor cells [17, 33–35]. Paauwe et al. [33] research showed that endoglin-expressing CAFs promoted colorectal cancer progression and metastasis. Dong et al. [34] explored the relationship between CAFs and bladder cancer cell lines by simulating the tumor microenvironment and using a co-culture system. Their study indicated that CAFs could regulated the invasion and metabolism of bladder cancer via autophagy. In addition, through the study of microRNAs, Lee et al. [35] found that CAFs promoted the migration and invasion of lung cancer via activated by miR-196a. Fourthly, CAFs induced tumor drug resistance during chemotherapy [18, 19, 36–39]. Qiao et al. [39] found that IL6 released by CAFs upregulated the expression of C-X-C motif chemokine receptor 7 via transcription 3/nuclear factor-kappa B signaling pathway, inducing chemotherapy resistance in esophageal squamous cell carcinoma. In gastric cancer, Zhang et al. [40] found that cisplatin and paclitaxel promoted the secretion of miR-522 from CAFs by activating the USP7/hnRNPA1 axis, and ultimately lead to chemotherapy resistance by targeting ALOX15 and blocking the accumulation of lipid ROS in cancer cells. Through the study of cell-surface molecules, Su et al. [6] found that CAFs subsets with CD10(+) GPR77(+) could promote tumor formation and chemotherapy resistance by providing niches for cancer stem cells. In a word, CAFs could induce the occurrence of tumor drug resistance by secreting various cytokines, exosomes and sustaining cancer stemness [41]. Finally, CAFs acted as a barrier to drug delivery into tumor cells [42]. As an important part of the extracellular matrix, CAFs were mainly distributed outside the tumor or around tumor vascular endothelial cells, forming a physical barrier to prevent drugs from entering tumor cells [42, 43]. Additionally, CAFs interacted with tumor cells by secreting a variety of cytokines, changing the tumor microenvironment and weakening the original effect of targeted drugs, thus acting as a biological barrier. As shown in Fig. 8A, CAFs play a crucial role in the tumor microenvironment and are an indispensable part. In-depth study of CAFs will help to further analyze the mechanism of tumorigenesis and development, and provide a potential theoretical basis for tumor therapy, especially immunotherapy.

Recent studies have shown that exosomes, as a new type of extracellular vesicles, have the function of intercellular communication in tumor microenvironment [44–46]. They are tiny vesicles with lipid bilayer membrane, which can be secreted by many cells. Exosomes often carry a variety of biological information, such as protein, lipid, DNA and RNA [45]. After being ingested by recipient cells, exosomes can trigger a variety of biological information exchange and complete cell-to-cell information transmission, thus affecting the physiological state of recipient cells and participating in the occurrence and progress of diseases [44, 47]. CAFs can affect the pathological process of tumors and the resistance of tumor cells to chemotherapy through the secretion of exosomes. Jiang et al. [48] found that CAFs-derived exosomes reduced cell apoptosis and promoted lung cancer progression by reducing miR-142-5p and upregulating PD-L1. In the study of malignant lymphoma, Kunou et al. [49] claimed that CAFs-derived exosomes induced anti-pyrimidine drug resistance via modulating the pyrimidine transporter, equilibrative nucleoside transporter 2. Interesting research by Liu et al. [50] found that CAFs-derived exosomes elicited radioresistance via promoting cancer stem cells in colorectal cancer. Furthermore, studies also had shown that CAFs-derived exosomes mediated tumor cell proliferation, invasion, metastasis, and angiogenesis [14, 30, 51, 52]. Scholars are becoming more and more interested in the mechanisms and functions of exosomes. As shown in Fig. 8B, there should be more papers published in this field in coming years. Therefore, with further research, CAFs-derived exosomes may provide new ideas for the treatment of tumors.

In recent years, tumor immunotherapy has been an unprecedented development, which has rapidly changed the prospect of tumor treatment [53–55]. The newly proposed tumor immune microenvironment (TIME) was closely related to the clinical prognosis of patients [1]. CAFs play an important role in TIME by interacting with immune cells to reshape TIME. There has been a substantial body of research demonstrating the importance of the interaction between CAFs and immune cells, especially tumor infiltrating lymphocytes cells (TILs) [56–59], tumor-associated macrophages [60–62] and natural killer cell [63–65]. Kato et al. [59] showed that CAFs could regulate TILs in patients with esophageal cancer, specifically by influencing CD8(+) and FoxP3(+) T cells through IL-6 in TME. At the same time, they also found that CD8(+) TILs were negatively correlated with CAFs. In breast cancer, Hu et al. [58] demonstrated that IL6 derived from CAFs could differentiate into regulatory T cells (Treg) through the IL6/STAT3 pathway, that is, CD73(+) gamma delta Tregs, which has a more powerful immunosuppressive effect and promoted tumor progression. Studies had shown that CAFs and tumor macrophages have a synergistic effect [63, 66]. CAFs could promoted the recruitment of monocytes to tumor and induced their differentiation to M2 macrophages, while M2 macrophages could affect the mesenchymal–mesenchymal transformation of fibroblasts, resulting in enhanced the vitality of tumor cells and ultimately promoted the spread and metastasis and neovascularization [62, 66]. In addition, the study of Ziani et al. [64] showed that CAFs were closely related to natural killer cell, and CAFs could reduce the sensitivity of tumor cells to NK cell-mediated lysis by secreting matrix metalloproteinases. In brief, CAFs were inextricably linked with immune cell regulation and had complex mechanisms, which may provide new ideas for tumor immune targeted therapy after in-depth study.

### Limitations

It is worth noting that, the study's data were exclusively gathered from WoSCC, potentially neglecting pertinent information available in other databases (ie. Scopus, PubMed), there are other limitations that should be taken into account, such as, new research is advancing rapidly, and groundbreaking studies that have been recently published may be missed. In addition, when establishing prediction models, it is necessary to consider that models may need to be adjusted for different regions, time periods, and economic development situations, otherwise bias may be introduced and the predictive results may not be accurate enough.

## Conclusions

This is the first thoroughly scientific bibliometric analysis and visualized study of the global research field on CAFs over the past 20 years. Apart from China, Western developed countries, especially the USA, are the major contributors in this field. The journal *Cancers* is the most popular journal for contributors in the area. At present and even coming years, CAFs may play an increasingly significant role in immunotherapy and tumor microenvironment, and more and more studies will be published. In conclusion, the study may provide benefits for researchers to master CAFs' dynamic evolution and research trends.

### Supplementary Information

**Additional file 1: Figure S1.** (A) and (B) Model logistic fitting curve of global trends in publications related to CAFs of developed country. (C) and (D) Model logistic fitting curve of global trends in publications related to CAFs of developing country.

**Additional file 2: Figure S2.** (A) Mapping of keywords in the research related to CAFs research from 1999–2021 (USA). (B) Distribution of keywords according to the mean frequency of appearance; keywords in yellow appeared later than those in blue (USA). (C) Mapping of keywords in the research related to CAFs research from 1999–2021 (China). (D) Distribution of keywords according to the mean frequency of appearance; keywords in yellow appeared later than those in blue (China); (E) Mapping of keywords in the research related to CAFs research from 1999–2021 (Japan). (F) Distribution of keywords according to the mean frequency of appearance; keywords in yellow appeared later than those in blue (Japan).

**Additional file 3: Figure S3.** (A) Mapping of keywords in the research related to CAFs research from 1999–2021 (University of Texas System). (B) Distribution of keywords according to the mean frequency of appearance; keywords in yellow appeared later than those in blue (University of Texas System). (C) Mapping of keywords in the research related to CAFs research from 1999–2021 (Institut National de la Sante et de la Recherche Medicale INSERM). (D) Distribution of keywords according to the mean frequency of appearance; keywords in yellow appeared later than those in blue (Institut National de la Sante et de la Recherche Medicale INSERM); (E) Mapping of keywords in the research related to CAFs research from 1999–2021 (University of California System). (F) Distribution of keywords according to the mean frequency of appearance; keywords in yellow appeared later than those in blue (University of California System).

## Data Availability

The original data presented in the study are included in the article and Supplementary Digital Content. Further inquiries can be directed to the corresponding author.
