# Supplementary figures and images for: Global cluster analysis and network visualization in cancer-associated fibroblast: insights from Web of Science database from 1999 to 2021
Source: Eur J Med Res. 2023 Nov 29;28:549. doi: 10.1186/s40001-023-01527-3 (PMC10685623; doi:10.1186/s40001-023-01527-3)

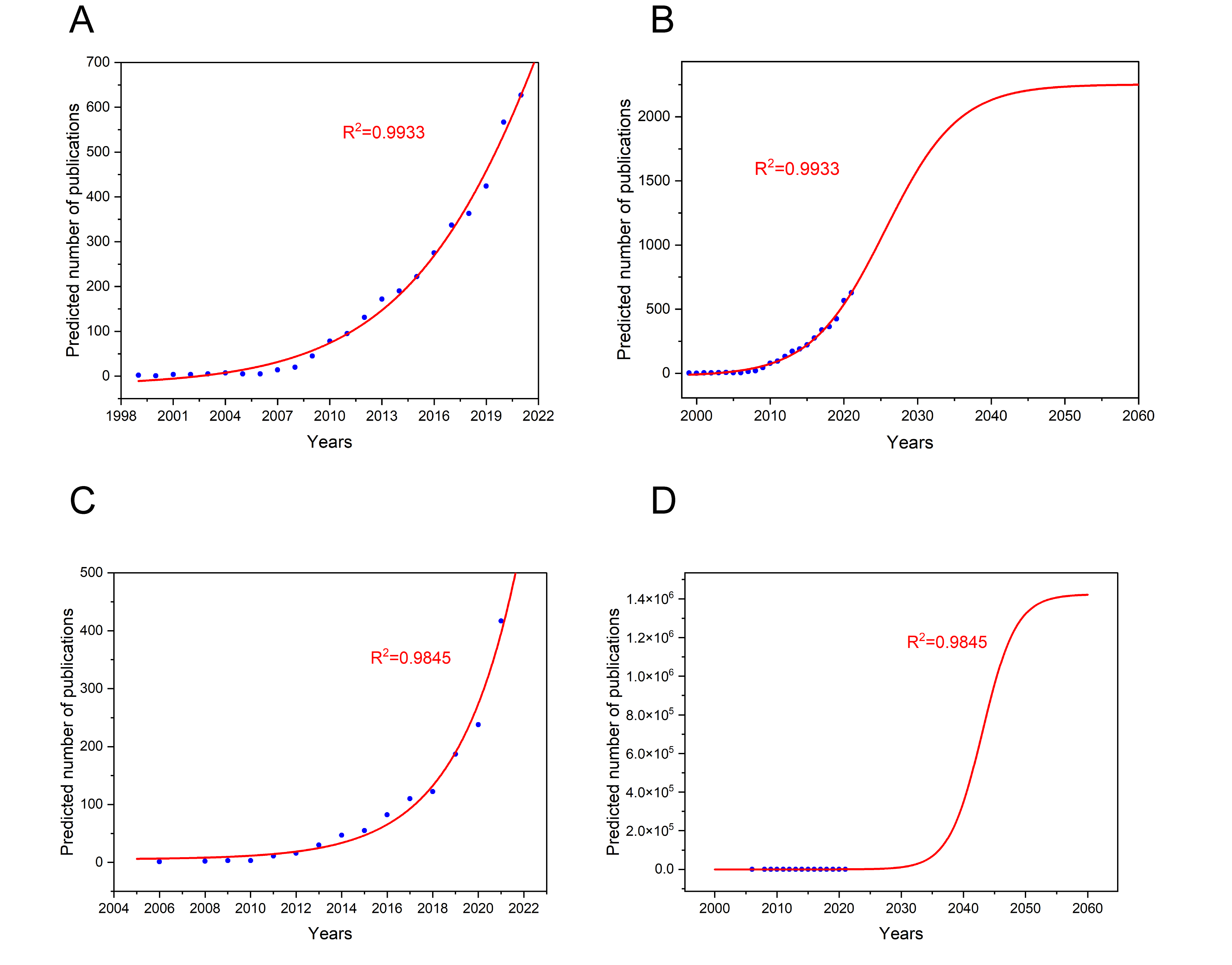

Supplement: Supplementary file 1 — Additional file 1: Figure S1. (A) and (B) Model logistic fitting curve of global trends in publications related to CAFs of developed country. (C) and (D) Model logistic fitting curve of global trends in publications related to CAFs of developing country. [file 40001_2023_1527_MOESM1_ESM.tif]

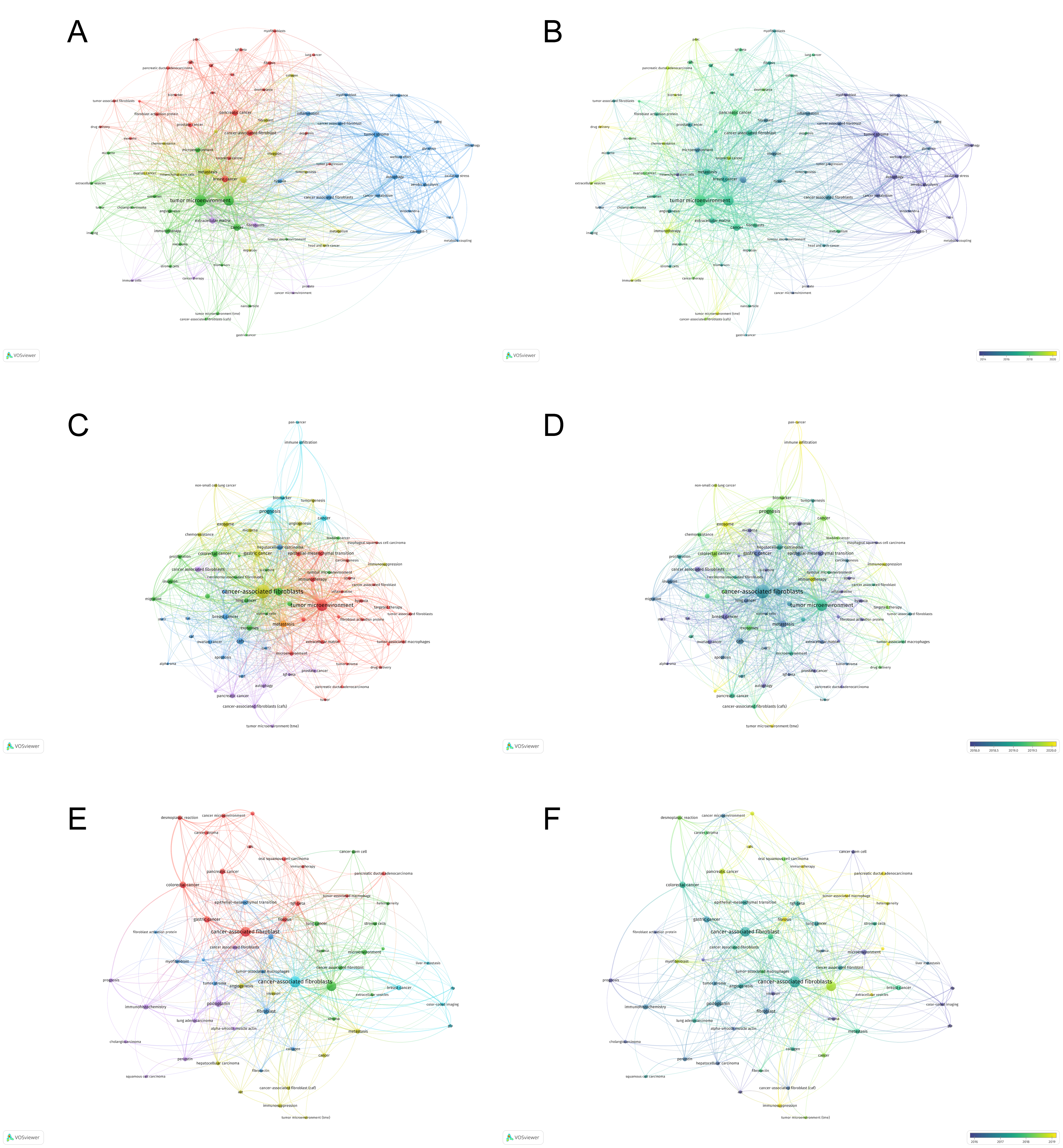

Supplement: Supplementary file 2 — Additional file 2: Figure S2. (A) Mapping of keywords in the research related to CAFs research from 1999–2021 (USA). (B) Distribution of keywords according to the mean frequency of appearance; keywords in yellow appeared later than those in blue (USA). (C) Mapping of keywords in the research related to CAFs research from 1999–2021 (China). (D) Distribution of keywords according to the mean frequency of appearance; keywords in yellow appeared later than those in blue (China); (E) Mapping of keywords in the research related to CAFs research from 1999–2021 (Japan). (F) Distribution of keywords according to the mean frequency of appearance; keywords in yellow appeared later than those in blue (Japan). [file 40001_2023_1527_MOESM2_ESM.tif]

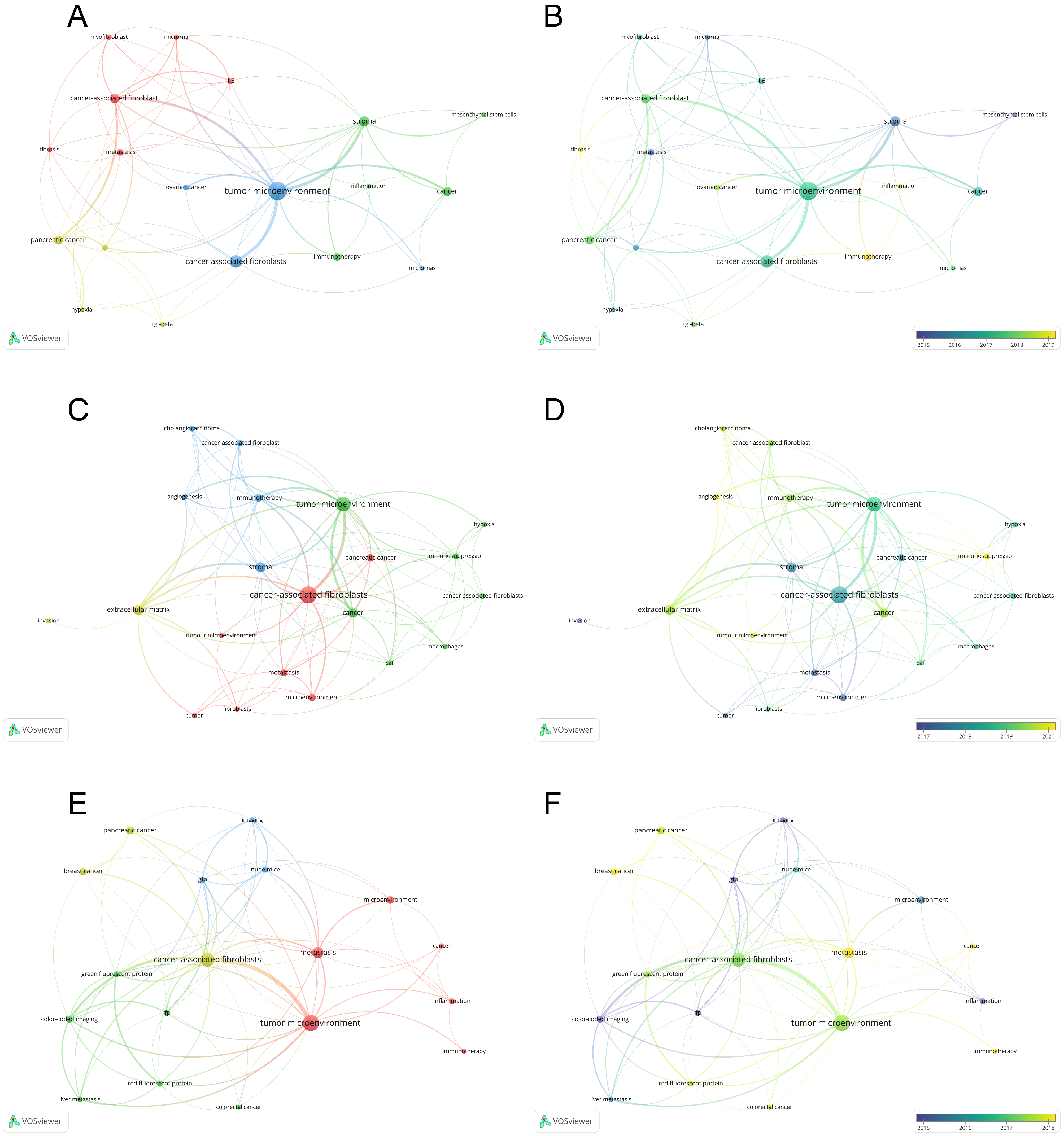

Supplement: Supplementary file 3 — Additional file 3: Figure S3. (A) Mapping of keywords in the research related to CAFs research from 1999–2021 (University of Texas System). (B) Distribution of keywords according to the mean frequency of appearance; keywords in yellow appeared later than those in blue (University of Texas System). (C) Mapping of keywords in the research related to CAFs research from 1999–2021 (Institut National de la Sante et de la Recherche Medicale INSERM). (D) Distribution of keywords according to the mean frequency of appearance; keywords in yellow appeared later than those in blue (Institut National de la Sante et de la Recherche Medicale INSERM); (E) Mapping of keywords in the research related to CAFs research from 1999–2021 (University of California System). (F) Distribution of keywords according to the mean frequency of appearance; keywords in yellow appeared later than those in blue (University of California System). [file 40001_2023_1527_MOESM3_ESM.tif]
